# Identification of age‐ and immune‐related gene signatures for clinical outcome prediction in lung adenocarcinoma

**DOI:** 10.1002/cam4.6330

**Published:** 2023-07-11

**Authors:** Andrew Zhou, Dalin Zhang, Xiaoman Kang, James D. Brooks

**Affiliations:** ^1^ Department of Urology Stanford University School of Medicine Stanford California USA; ^2^ Department of Oncology Stanford University School of Medicine Stanford California USA

**Keywords:** age, immune infiltration, lung adenocarcinoma, overall survival, tumor microenvironment

## Abstract

**Background:**

The understanding of the factors causing decreased overall survival (OS) in older patients compared to younger patients in lung adenocarcinoma (LUAD) remains.

**Methods:**

Gene expression profiles of LUAD were obtained from publicly available databases by Kaplan‐Meier analysis was performed to determine whether age was associated with patient OS. The immune cell composition in the tumor microenvironment (TME) was evaluated using CIBERSORT. The fraction of stromal and immune cells in tumor samples were also using assessed using multiple tools including ESTIMATE, EPIC, and TIMER. Differentially expressed genes (DEGs) from the RNA‐Seq data that were associated with age and immune cell composition were identified using the R package DEGseq. A 22‐gene signature composed of DEGs associated with age and immune cell composition that predicted OS were constructed using Least Absolute Shrinkage and Selection Operator (LASSO).

**Results:**

In The Cancer Genome Atlas (TCGA)‐LUAD dataset, we found that younger patients (≤70) had a significant better OS compared to older patients (>70). In addition, older patients had significantly higher expression of immune checkpoint proteins including inhibitory T cell receptors and their ligands. Moreover, analyses using multiple bioinformatics tools showed increased immune infiltration, including CD4+ T cells, in older patients compared to younger patients. We identified a panel of genes differentially expressed between patients >70 years compared to those ≤70 years, as well as between patients with high or low immune scores and selected 84 common genes to construct a prognostic gene signature. A risk score calculated based on 22 genes selected by LASSO predicted 1, 3, and 5‐year OS, with an area under the curve (AUC) of 0.72, 0.72, 0.69, receptively, in TCGA‐LUAD dataset and an independent validation dataset available from the European Genome‐phenome Archive (EGA).

**Conclusion:**

Our results demonstrate that age contributes to OS of LUAD patients atleast in part through its association with immune infiltration in the TME.

## 1 INTRODUCTION

Non‐small cell lung cancer (NSCLC) accounts for approximately 85% of the estimated 130,180 deaths due to lung cancer in the United States in 2022.^1^ Lung adenocarcinoma (LUAD), the most common histological subtype of NSCLC, is a genetically heterogeneous disease that can be classified into molecular subtypes based on mutational drivers including tyrosine kinases (EGFR, ALK, ROS1, NTRK, RAF, MET, and RET) that can be targeted rationally using the 17 FDA‐approved tyrosine kinase inhibitors (TKIs), as well as KRAS, a common NSCLC molecular subtype with limited treatment options currently.^2^, ^3^ Recently, immune checkpoint inhibitor (ICI)‐based immunotherapy that enhances T‐cell activation to eliminate cancer cells has become an indispensable second‐line therapy for NSCLC patients with innate and required resistance to TKIs, and as first‐line therapy for those lacking targetable driver mutations.^4^, ^5^, ^6^ With these recent additions to the treatment landscape of NSCLC, the 5‐year overall survival (OS) for patients with metastatic disease has improved from below 5% to more than 30% with TKIs, and 20% for ICIs.^7^, ^8^, ^9^ Accurate identification of patients with poor prognosis that require aggressive treatment could guide treatment selection and further improve the outcomes of NSCLC patients.

One of the approaches to classify tumors into relevant prognostic groups is to develop prognostic gene expression signatures taking advantages of multi‐omics technologies.^10^, ^11^ Such gene expression signatures have been developed, validated, and implemented in clinical settings for multiple cancer types including breast cancer (BCa), prostate cancer (PCa), colon cancer, and thyroid cancer.^12^, ^13^, ^14^ Moreover, gene expression signatures associated with specific patient characteristics (e.g., age) and features such as the tumor microenvironment (TME) have been found to be associated with worse survival in several cancers, consistent with their significant contributions to cancer progression.^15^, ^16^, ^17^, ^18^ For example, in The Cancer Genome Atlas‐Prostate Adenocarcinoma (TCGA‐PRAD) cohort, the age‐ and TME‐related risk score (ATRS), developed using gene expression profiles, was associated with the expression of immune checkpoint proteins and immune cytolytic activities of the intratumoral immune cell infiltration in PCa.^19^ In addition, an ATRS‐based nomogram performed well in predicting the outcomes of PCa patients.^19^ Moreover, in a pan‐cancer transcriptome analysis using TCGA data, patients with advanced age (>75 years old) not only showed worse OS compared to young patients (<50 years old) in 16 cancer types, including LUAD, but also exhibited unique gene expression changes affecting multiple important pathways implicated in cancer progression including epithelial–mesenchymal transition (EMT), KRAS signaling, p53 pathways, and hypoxia.^20^ Finally, advanced age was associated with significantly increased numbers of innate immune cell infiltrates and upregulation of immune checkpoint proteins including PD‐1, PD‐L1, PD‐L2, and CTLA‐4, creating a more immune suppressive TME compared to young patients.^20^ However, whether age and TME‐related gene expression predicts survival for patients with LUAD, and the impact of aging on the TME, particularly immune cell infiltrates and immune checkpoint protein expression, has not been investigated.

In this study, we analyzed gene expression profiles of LUAD in TCGA to determine whether age was associated with patient OS by Kaplan–Meier analysis.^21^ Next, we compared the immune cell composition in the TME of older (>70 years) versus younger (≤70 years) patients using CIBERSORT, a robust method for characterizing cell composition of complex tissues based on their gene expression profiles.^22^ In addition, we inferred the fraction of stromal and immune cells in tumor samples using multiple tools including ESTIMATE (Estimation of STromal and Immune cells in MAlignant Tumors using Expression data),^23^ EPIC (Estimating the Proportions of Immune and Cancer cells),^24^ and TIMER (Tumor IMmune Estimation Resource).^25^ Furthermore, we identified differentially expressed genes (DEGs) from the RNA‐Seq data that were associated with age and immune cell composition using the R package DEGseq.^26^ Finally, we established a 22‐gene signature composed of DEGs associated with age and immune cell composition that predicted OS in TCGA‐LUAD and validated this predictor in independent LUAD datasets available from the European Genome‐phenome Archive (EGA).

## 2 MATERIALS AND METHODS

### 2.1 TCGA‐LUAD expression dataset

The gene expression profiles determined by RNAseq of 517 TCGA‐LUAD patients with associated clinical data were downloaded through the Genome Data General Database (GDC) data portal (https://gdac.broadinstitute.org/), out of which 498 with age information were used for data analysis.

### 2.2 Survival analysis stratified by age

The TCGA‐LUAD patients were divided into two age groups (≤70, >70 years) and the survival differences were determined by the Log‐rank (Mantel‐Cox) test using GraphPad Prism 8 (La Jolla, CA). A value of *p* < 0.05 was considered statistically significant.

### 2.3 CIBERSORT score and immune checkpoint gene expression

Immune cell infiltration and immune checkpoint gene expression were analyzed in different age groups using the online bioinformatics portal (https://www.aclbi.com/) in accordance with guidelines available at the site. Briefly, the CIBERSORT^22^ method was applied to TCGA‐LUAD patient samples to generate a CIBERSORT score. The CIBERSORT scores were compared for patients of low age (≤70) versus high age (>70). Similarly, expression levels of immune checkpoint genes were compared between these two age groups using the immunity analysis module.

### 2.4 Correlation of age and survival with the immune/estimate/stromal score

The immune algorithm ESTIMATE^23^ was utilized to assess tumor purity, the relative level of stromal cells, and the amount of immune cell infiltration in TCGA‐LUAD based on expression data between the low age (≤70) and high age (>70) groups (https://bioinformatics.mdanderson.org/estimate/). The stromal and immune scores determined using ESTIMATE are based on gene expression. Briefly, ESTIMATE outputs stromal, immune and ESTIMATE scores by performing ssGSEA (single‐sample gene set enrichment analysis). For a given sample, gene expression values are first rank‐normalized and rank‐ordered. Then the empirical cumulative distribution functions of the genes in the signature and the remaining genes are calculated. Finally, a statistical score is calculated by an integration of the difference between the empirical cumulative distribution function, which is similar to that used in gene set‐enrichment analysis but based on absolute expression rather than differential expression. The statistical scores based on the signatures related to stromal tissue and immune cell infiltration are designated as stromal and immune scores and these are combined to generate the ESTIMATE score. The Immune/Estimate/Stromal scores were tested for their relationships with age and survival using Pearson correlation in GraphPad 8.0.

### 2.5 Estimation of the proportion of cell types from bulk gene expression data by EPIC

We evaluated the cell compositions of tumor tissues in TCGA‐LUAD patients using EPIC^24^ (https://rdrr.io/github/GfellerLab/EPIC/man/EPIC.html). Fractions of different cell types calculated using EPIC were evaluated for associations with age and survival by Pearson correlation in GraphPad 8.0.

### 2.6 Immune infiltration analysis using TIMER and proteomics data

The proteomics dataset PDC000219^27^ containing 103 LUAD patients with associated clinical information and PDC000153^28^ containing 102 LUAD with clinical data were obtained from the proteomic data commons (https://pdc.cancer.gov/pdc/). The average expression of each protein was calculated and log2 transformed to adapt to the TIMER estimation tools.^25^ The infiltration of different immune cell types in patients of low age (≤70) and high age (>70) was analyzed using the TIMER estimation module.^25^

### 2.7 Identification of DEGs associated with age and/or immune score in LUAD

The “DEseq2” R package was employed to identify DEGs between patients of low age (≤70) versus high age (>70) as well as patients with low immune scores versus high immune scores in TCGA‐LUAD. We required that DEGs used in the analysis were expressed in at least half of the patient samples, |log2FC| ≥ 1 and *p*Value ≤ 0.01. The “EnhancedVolcano, ggplot2, and ggrepel” R packages were employed for generating the Volcano plot. DEGs associated with age and/or immune scores were displayed as Venn diagrams using the functional enrichment analysis tool.^29^

### 2.8 Pathway enrichment analysis

Pathway enrichment analysis was performed using DEGs and Database for Annotation, Visualization, and Integrated Discovery (DAVID; http://www.david.niaid.nih.gov).^30^ The top significantly enriched pathways with *p*‐adj < 0.05 were plotted.

### 2.9 Construction of the prognostic gene signature and the risk score

Common genes that are differentially expressed between patients of low age (≤70) and high age (>70 years) as well as between patients with high versus low immune scores from TCGA‐LUAD were used to develop a prognostic signature using the LASSO (Least Absolute Shrinkage and Selection Operator) model in glmnet package.^31^, ^32^ A risk score was calculated using the following formula based on the expression levels of 22 genes selected by LASSO: Riskscore = (0.062) * SPIB + (0.2452) * ABCC8 + (0.0271) * CNGA3 + (0.0155) * CPLX2 + (0.2609) * DRP2 + (0.0122) * FGA + (0.0097) * GUCY2C + (0.0055) * IL17C + (0.0455) * INHA + (−0.0285) * KLK13 + (−0.0844) * MTMR7 + (−0.0563) * MYO18B + (0.0915) * NEFL + (−0.0485) * NOTUM + (0.0211) * PCK1 + (0.0191) * SCG2 + (−2e‐04) * SCN4A + (−0.0328) * TAC4 + (0.0065) * VGF + (−1e‐04) * BPIFB2 + (−0.0269) * STPG3 + (−0.0192) * NEURL1.

## 3 RESULTS

### 3.1 Age is associated with poor prognosis and immunosuppressive TME in LUAD

**FIGURE 1:**
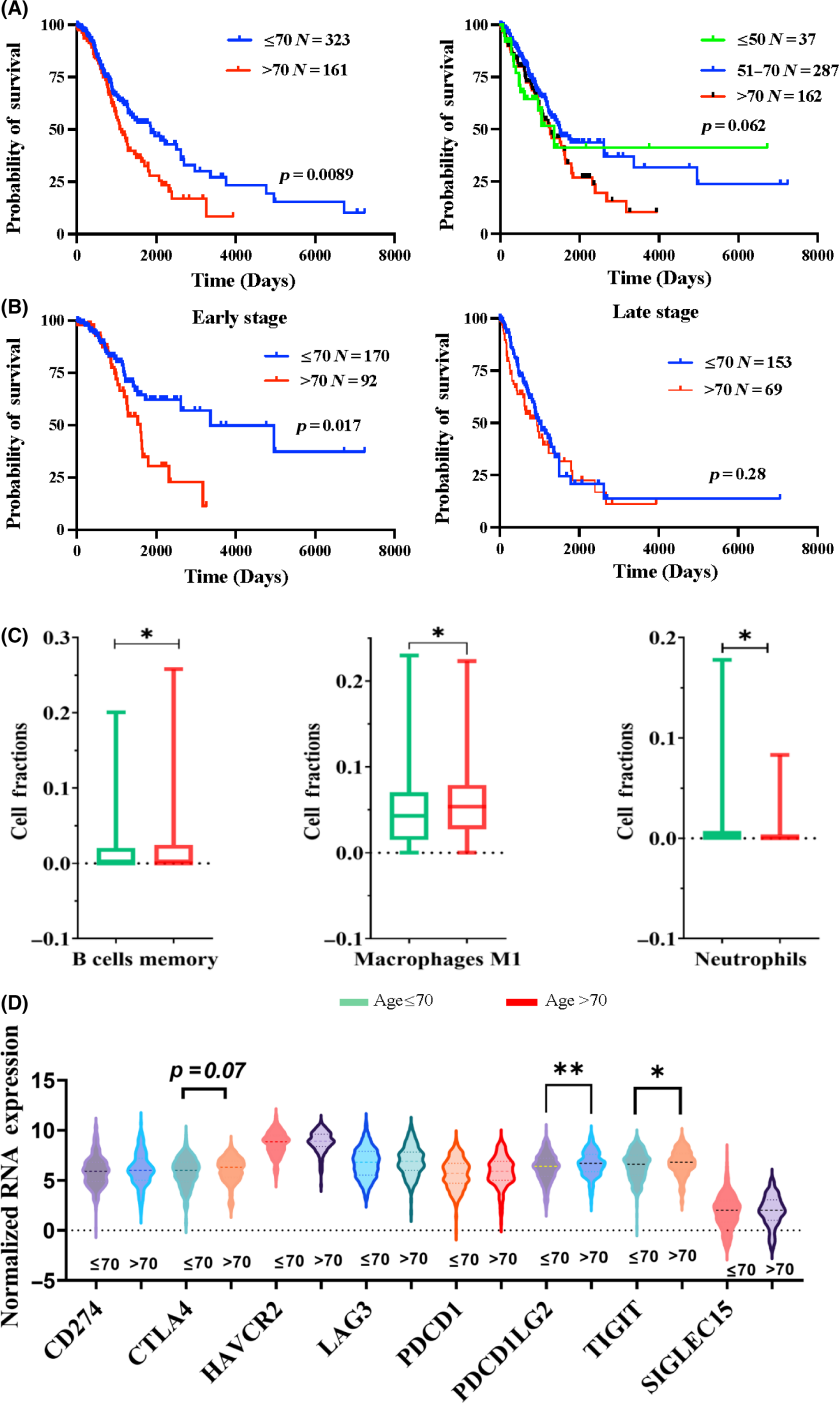
Age is associated with poor prognosis and immunosuppressive tumor microenvironment in lung adenocarcinoma (LUAD). (A) Kaplan–Meier plot of The Cancer Genome Atlas‐Prostate Adenocarcinoma‐lung adenocarcinoma (TCGA‐LUAD) patients divided into different age groups. (B) Survival analysis of TCGA‐LUAD patients with early‐ or late‐stage disease divided into different age groups. (C) Infiltration of different immune subtypes in TCGA‐LUAD patients >70 years compared to those ≤70 years. (D) Expression of immune checkpoint genes in TCGA‐LUAD patients >70 years compared to those ≤70 years. *p < 0.05, **p < 0.01.

To determine the prognostic value and immune influences of age in LUAD, we performed survival analysis using the Log‐rank (Mantel‐Cox) test on TCGA‐LUAD patients. Patients whose ages were ≤70 years had significantly better overall survival (OS) compared to patients >70 years (*p* = 0.0089) (Figure 1A). Survival differences among patients that were ≤50, 51–70 and >70 years also showed a trend of inverse correlation of age and survival that approached statistical significance (*p* = 0.062) (Figure 1A). The small sample size of patients ≤50 years (*N* = 37) might have contributed to the lack of statistical significance in the survival analysis. In addition, in early‐stage (stage 1) disease, younger patients (≤70) had significantly improved OS compared to older patients (>70), while in late‐stage (stage 2, 3, and 4) disease, no differences in OS were detected between patients in the two age groups (Figure 1B).

Interestingly, CIBERSORT^22^ analysis revealed that patients aged >70 years showed higher immune infiltration of memory B cells, M1 macrophages, and lower immune infiltration of neutrophils compared to patients whose age was ≤70 years (Figure 1C). Moreover, these patients had significantly higher expression of immune checkpoint proteins, including the inhibitory T‐cell receptor TIM3 (T cell immunoglobulin and mucin domain‐containing protein 3, also known as TIGIT),^33^ and PD‐L2 (also known as PDCDILG2), an alternate ligand for PD‐1 that also inhibits T‐cell activation^34^ (Figure 1D). CTLA4, another inhibitory T‐cell receptor,^35^ showed a trend toward increased expression in older patients compared to younger patients (Figure 1D). Taken together, these results suggest that LUAD in patients >70 years displayed a more immunosuppressive TME compared to younger patients.

### 3.2 Age is positively associated with immune infiltration in LUAD

**FIGURE 2:**
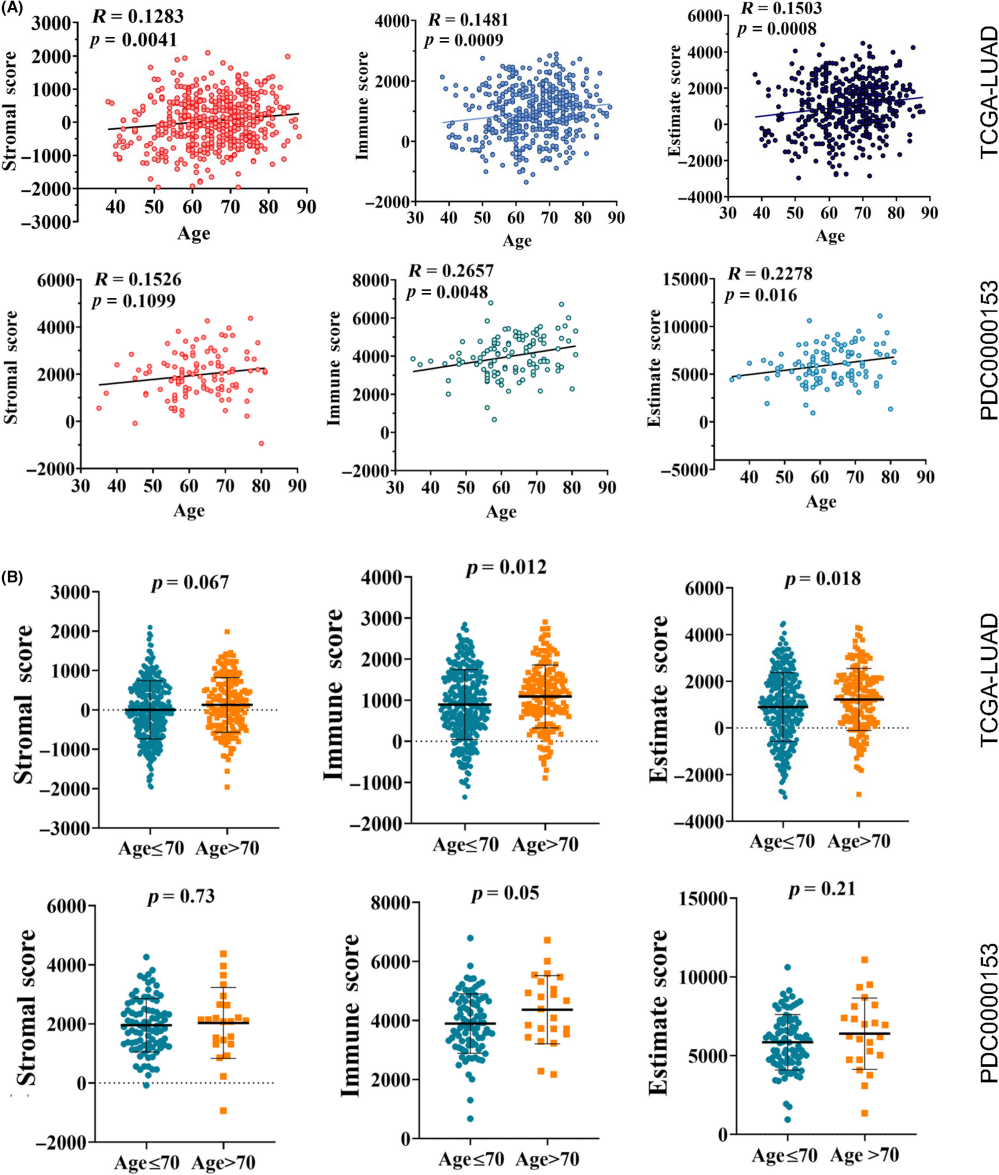
Age is positively associated with immune infiltration in lung adenocarcinoma (LUAD). (A) Pearson correlation analysis demonstrated that age is positively associated with tumor purity measured by estimate score and immune infiltration measured by immune score in both The Cancer Genome Atlas‐Prostate Adenocarcinoma‐LUAD and PDC000153 datasets. (B) Patients aged >70 years had significantly higher immune infiltration with no quantitative differences in stromal cells compared to younger patients in both datasets.

To determine the effects of patient age on cell type compositions of the tumors in LUAD, we calculated the estimate score, stromal score, and immune score using ESTIMATE which represents tumor purity, the level of stromal cells present, and the level of immune cell infiltrates. As shown in Figure 2A, Pearson correlation analysis demonstrated that age is positively associated with tumor purity and immune infiltration in both TCGA‐LUAD and PDC000153 datasets. In addition, the correlation between age and the stromal score was significant in TCGA‐LUAD dataset but not in the PDC0000153 dataset, possibly due to the smaller sample size in the latter dataset. Moreover, patients >70 years had significantly higher immune infiltration on average, but did not show differences in the amount of stromal cells present or tumor purity compared to younger patients in both datasets (Figure 2B), suggesting that aging is associated with immune infiltration in LUAD tumor tissues.

### 3.3 Age is associated with changes in tumor cell composition in the TME of LUAD

**FIGURE 3:**
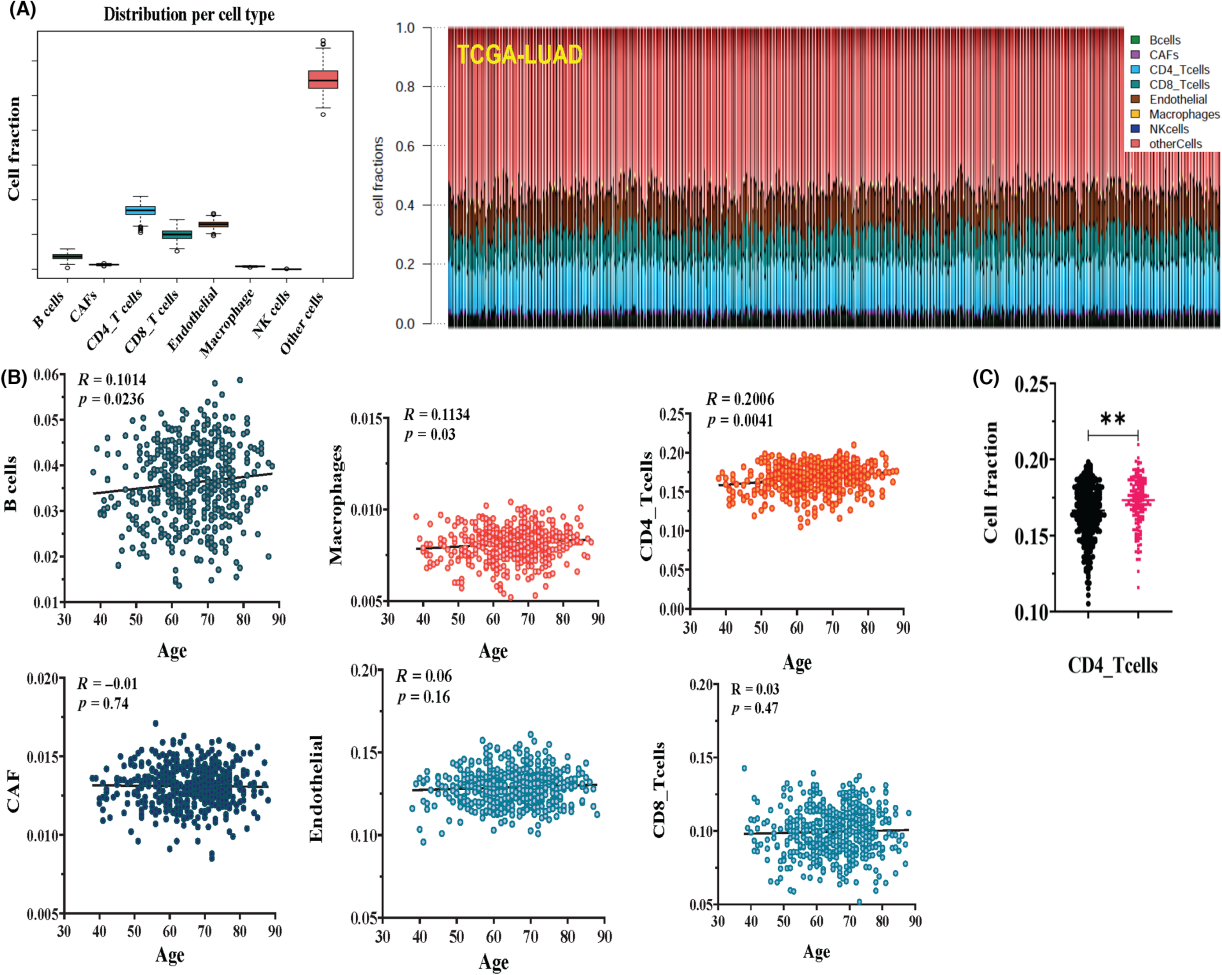
Age is associated with changes in cell compositions within tumor microenvironment (TME) in lung adenocarcinoma (LUAD). (A) Estimated proportions of eight cell types within TME in The Cancer Genome Atlas‐Prostate Adenocarcinoma‐LUAD using Estimating the Proportions of Immune and Cancer cells. (B) The percentages of CD4+ T cells, B cells, and macrophages were positively associated with age, while the fractions of CD8+ T cells, cancer associated fibroblasts, and endothelial cells were not. (C) The fractions of CD4+ T cells were significantly higher in patients >70 years compared to those ≤70 years. *p < 0.05, **p < 0.01.

We evaluated the cell composition of LUAD tissues in different age groups using multiple tools. First, we estimated the proportion of various cell types in the TME including immune cells, cancer associated fibroblasts (CAFs), and endothelial cells in TCGA‐LUAD using EPIC as described previously.^36^ Among the three common cell types, immune cells comprised the highest percentage in the TME of TCGA‐LUAD followed by endothelial cells and CAFs (Figure 3A). Among immune cells, CD4+ T cells were the most abundant type, CD8+ T cells were second most abundant, followed by B cells, macrophages, and natural killer cells (Figure 3A). In addition, the percentages of CD4+ T cells, B cells, and macrophages were positively associated with age, while the fractions of CD8+ T cells, CAFs, and endothelial cells were not significantly different among patients of different ages (Figure 3B). Consistently, fractions of CD4+ T cells were significantly higher in patients >70 years compared to those ≤70 (Figure 3C). Interestingly, immune infiltration was not significantly different in non‐smokers versus smokers in neither the TCGA‐LUAD dataset nor the PDC000153 dataset, regardless of age (Supplemental Figure 1). These results suggest that aging significantly influenced the cell composition of the TME in LUAD.

### 3.4 Comparing immune cell fractions determined using different tools within LUAD TME between age groups

**FIGURE 4:**
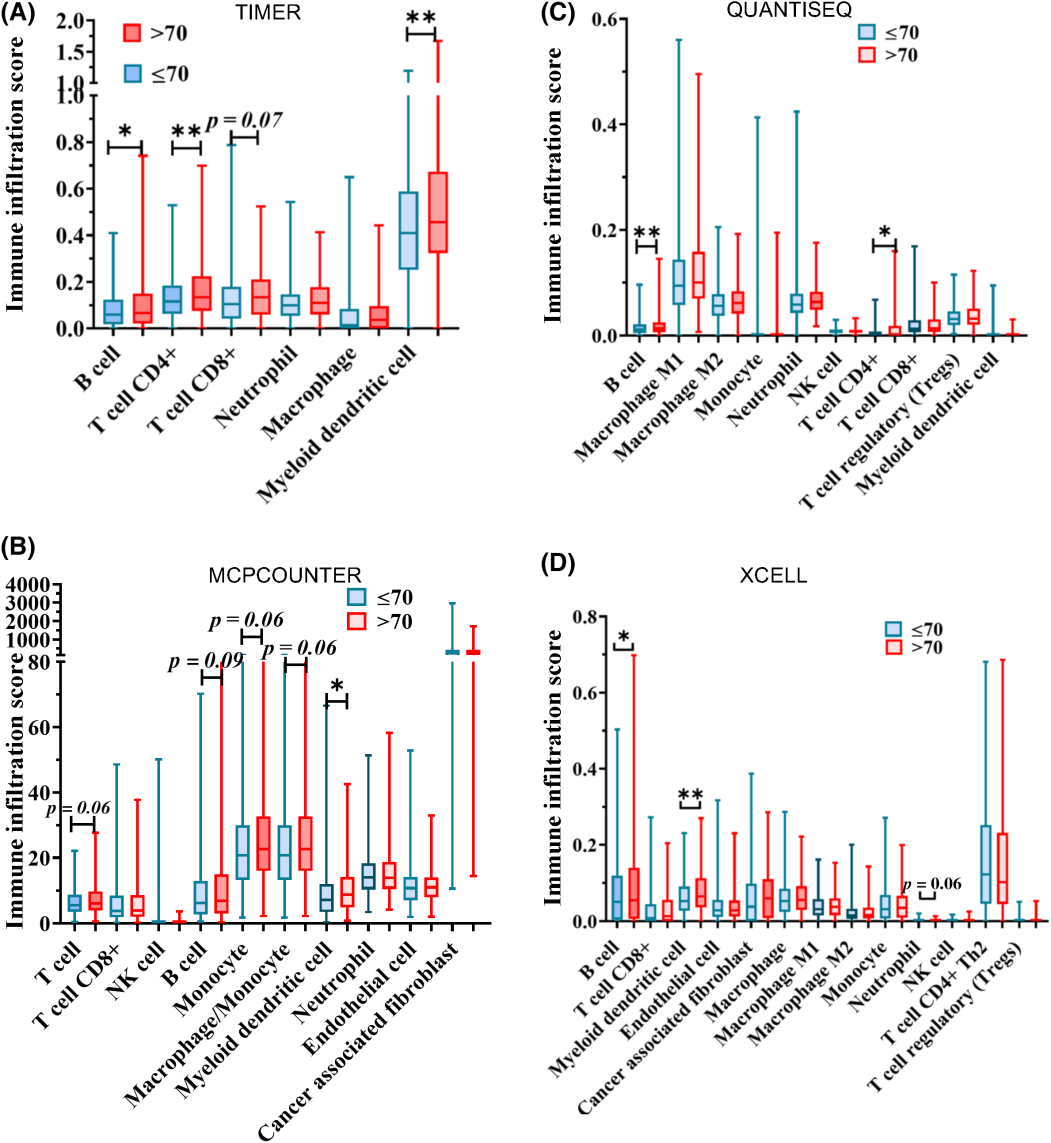
Comparison of immune cell fractions determined using different tools within lung adenocarcinoma tumor microenvironment (LUAD TME) between age groups. Timer (A), MCPCOUNTER (B), QUANTISEQ (C), and XCELL (D) were used to estimate immune cell infiltration within TME in The Cancer Genome Atlas‐Prostate Adenocarcinoma‐LUAD. *p < 0.05.

Multiple tools are available to evaluate immune cell fractions using gene expression datasets from bulk tumor tissues based on the levels of transcripts or proteins. To determine the degree of variation in the immune cell fractions extracted and annotated by these tools, we determined the relative amounts of immune cell subtypes using Timer, QUANTISEQ, XCELL, and MCPCOUNTER on the TCGA‐LUAD dataset (Figure 4) and compared the findings to CIBERSORT and EPIC (Table 1). The most consistent finding was that fractions of infiltrating CD4+ T cells were significant higher in patients >70 years compared to those ≤70 years (Figures 3C and 4A,C). Infiltrating B cells and mDC were consistently shown to be higher in patients >70 years compared to those ≤70 years using TIMER and QUANTISEQ, as well as MCPCOUNTER and XCELL (Figure 4). On the other hand, only CIBERSORT showed significant differences in infiltrating M1 macrophages and neutrophils that were not observed using the other tools (Figure 4 and Table 1). These results indicate that robust differences in infiltration of immune subtypes can be detected by multiple tools while subtle differences could vary between analysis platforms.

**TABLE 1:** Differences in immune cell fractions within lung adenocarcinoma tumor microenvironment (LUAD TME) between age groups (≤70 vs. >70) evaluated using multiple tools.

	CIBERSORT	EPIC	TIMER	QUANTISEQ	MCPCOUNTER	XCELL
B cell		NS	↑	↑	NS	NS
Memory B cell	↑					
CD4+ T cell	NS	↑	↑	↑		NS
CD8+ T cell	NS	NS	NS	NS	NS	NS
T cell					NS	
Treg				NS		NS
Macrophage		NS	NS			NS
M1 Macrophage	↑			NS		NS
M2 Macrophage	NS			NS		NS
MDC	NS		NS	NS	↑	↑
Monocyte	NS				NS	NS
Neutrophil	↓	NS	NS	NS	NS	NS
NK cell	NS	NS		NS	NS	NS
Endothelial	NS	NS			NS	NS
CAF	NS	NS			NS	NS

### 3.5 Genes involved in critical pathways are enriched in DEGs associated with age and/or immune infiltration

**FIGURE 5:**
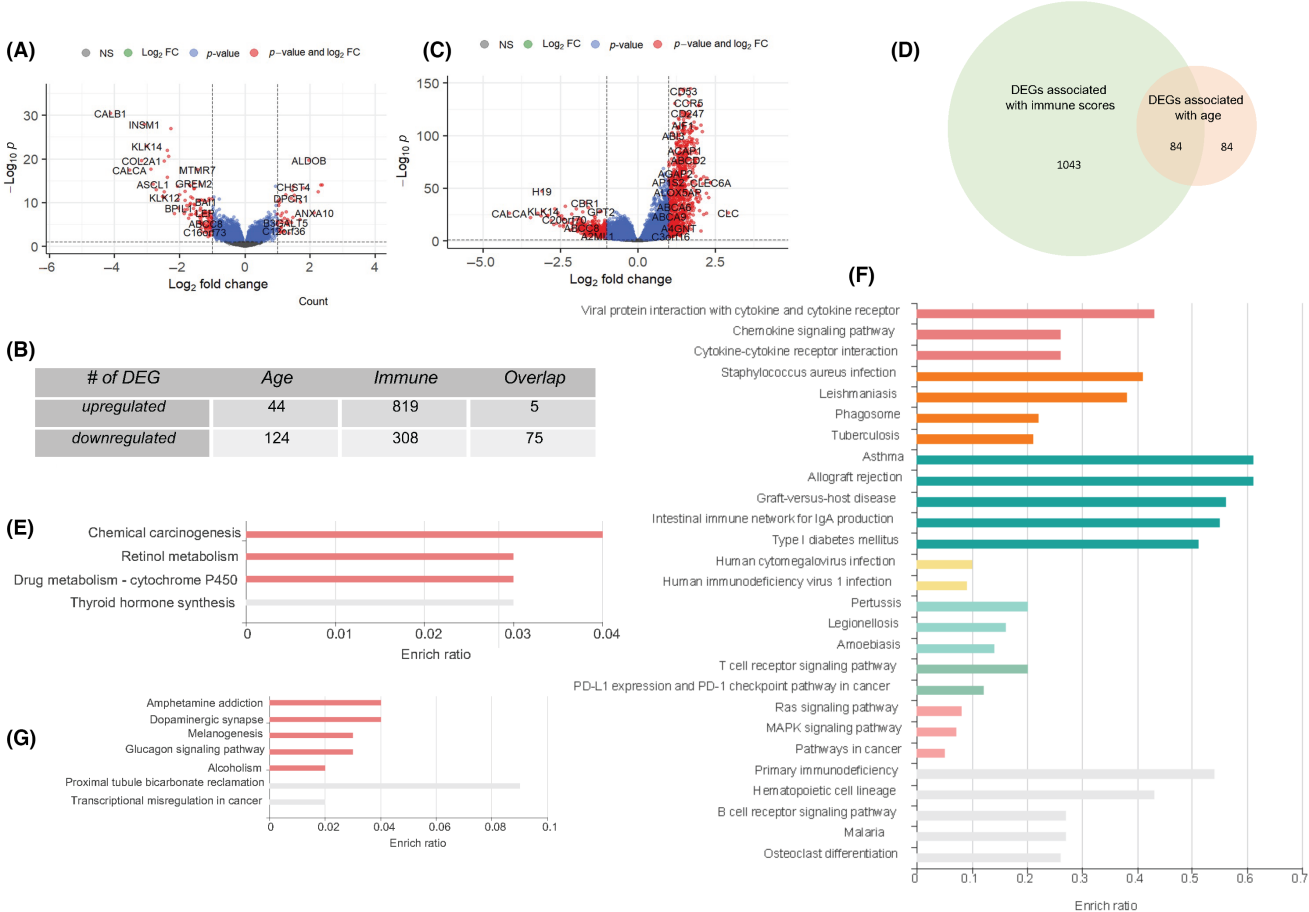
Genes involved in critical pathways are differentially expressed among patients in different age groups and/or with different immune infiltration. Volcano plots of differentially expressed genes (DEGs) between patients older than 70 versus 70 and younger (A) and patients with high versus low immune scores (C) in the Cancer Genome Atlas‐Prostate Adenocarcinoma‐lung adenocarcinoma dataset. The number of genes upregulated or downregulated in these two datasets as well as overlapping genes were listed in (B) and by Venn Diagram (D). The DEGs enriched pathways were shown by bubble plots for patients >70 versus ≤70 years (E) and patients with high versus low immune scores (F). Overlapping DEGs associated both age and immune infiltration shown were enriched in pathways shown in (G).

DEGs between patients >70 years compared to those ≤70 years in the TCGA‐LUAD dataset were identified using the “DEseq2” R package and plotted in Figure 5A. Forty‐four and 124 genes were upregulated or downregulated in patients >70 years compared to those ≤70 years (Figure 5B and Supplemental Table 1). Similarly, DEGs between tumors with low immune scores compared to those with high immune scores in the TCGA‐LUAD dataset were identified using the “DEseq2” R package and plotted in Figure 5C. Eight hundred and nineteen genes were upregulated and 308 genes were downregulated in tumors with high immune infiltration compared to those with low immune infiltration (Figure 5B and Supplemental Table 1), respectively. To identify common genes that are differentially expressed between patients >70 years compared to younger patients as well as between patients with high versus low immune scores, we selected 84 genes overlapping between the 168 and 1127 gene lists (Figure 5D and Supplemental Table 1). The direction of expression change (increased or decreased) for these 84 genes was consistent for 80 genes, with four genes showing expression changes in the opposite direction. Interestingly, the overlap between the upregulated genes was minimal (11.3% and 0.6% for age and immune, respectively), while downregulated genes overlapped significantly between the two lists (60.5% and 22.4% for age and immune, respectively).

Enrichment analysis showed that the top pathway enriched in age‐associated DEGs that were upregulated was chemical carcinogenesis (Figure 5E), suggesting that these genes may contribute to LUAD development and progression to a greater degree in older patients more than in younger patients. Among pathways enriched in upregulated immune‐associated DEGs were several pathways that confer decreased immune function, including PD‐L1 expression and the PD‐1 checkpoint pathway in cancer and primary immunodeficiency (Figure 5F), suggesting that the TME in older patients is more immunosuppressive than in younger patients. For genes overlapping between age and immune associated DEGs, including upregulated and downregulated genes, transcriptional dysregulation in cancer was identified as a significantly enriched pathway among others (Figure 5G), suggesting gene transcription may be a common biological process differentially affected in older versus younger LUAD patients.

### 3.6 Construction of a prognostic signature with DEGs associated with age and immune infiltration in LUAD

**FIGURE 6:**
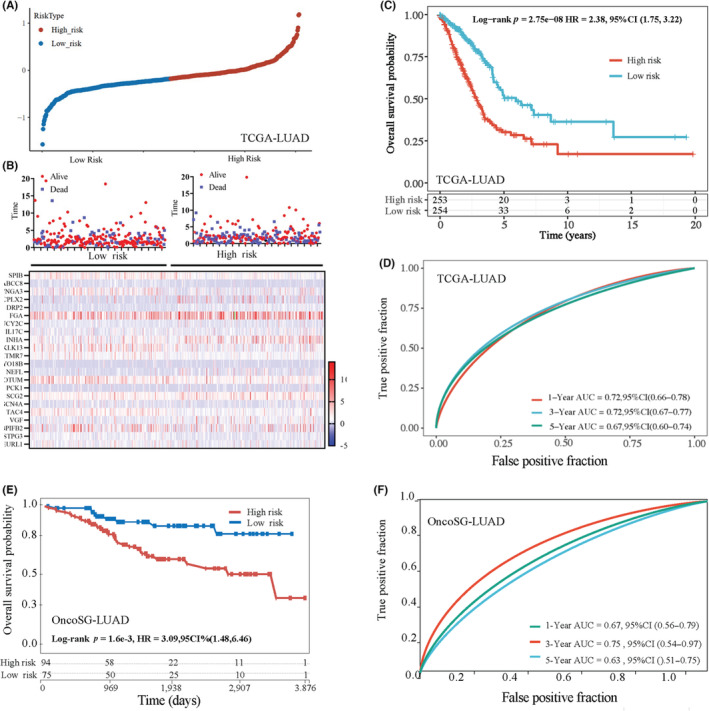
Construction of a prognostic signature with differentially expressed genes associated with age and immune infiltration in lung adenocarcinoma (LUAD). (A) A risk score was calculated using the weighted expression levels of the 22 genes and the median value was used as the cut‐off value to define high versus low risk. (B) The survival status of each LUAD patient and the expression levels of the 22 genes were shown in the heatmap. (C) The LUAD patients with higher risk scores had poorer overall survival (OS) than patients with lower risk scores in The Cancer Genome Atlas‐Prostate Adenocarcinoma(TCGA)‐LUAD dataset determined by Kaplan–Meier analysis. (D) The receiver operating characteristic (ROC) indicated that our prognostic signature performed well in predicting 1, 3, and 5‐year OS of TCGA‐LUAD patients, with an area under the curve (AUC) of 0.72, 0.72, 0.67, respectively. (E) The LUAD patients with higher risk scores had poorer OS than patients with lower risk scores in Onco_SG dataset determined by Kaplan–Meier analysis. (F) ROC curves indicated that our prognostic signature performed well in predicting 1, 3, and 5‐year OS of Onco_SG_LUAD patients, with an AUC of 0.72, 0.72, 0.67, respectively.

We developed a prognostic signature based on the expression levels of 22 genes selected by the LASSO model using glmnet package^31^, ^32^ out of the 84 common DEGs associated with age and immune scores in LUAD. A risk score was calculated using the weighted expression levels of the 22 genes and the median value was used as the cut‐off value to define high versus low risk (Figure 6A). The survival status of each LUAD patient and the expression levels of the 22 genes were shown in the heatmap (Figure 6B). The LUAD patients with higher risk scores had poorer OS than patients with lower risk scores determined by Kaplan–Meier analysis (Figure 6C). The receiver operating characteristic (ROC) curve indicated that our prognostic signature performed well in predicting 1‐, 3‐, and 5‐year OS, with an area under the curve (AUC) of 0.72, 0.72, 0.67, respectively (Figure 6D). We used several additional ML algorithms, including random forest, in an attempt to improve the performance of the predictive models. We also used additional approaches to model training and testing including 10‐fold cross validation and splitting data into training and test sets. However, LASSO outperformed all other algorithms, yielding higher AUCs than other approaches. For example, random forest generated AUCs for 1‐, 3‐, and 5‐year OS of 0.61, 0.644, and 0.60, respectively.

To validate our predictive gene signature, we downloaded transcriptional profiles of 305 LUAD patients of East Asian ancestry from the European Genome‐phenome Archive under accession codes EGAD00001004421 and EGAD00001004422 and are named OncoSG_LUAD dataset.^37^ Twenty out of the 22 predictive genes (except DRP2 and NEF) identified in our study were present in this dataset and were used to build a predictive model using LASSO similarly as for TCGA‐LUAD dataset. Risk scores were calculated and used to stratify patients into high risk and low risk groups. Patients with high risk scores showed significantly poorer OS compared to those with low risk scores (Figure 6E). The AUCs for 1‐, 3‐, and 5‐year OS for OncoSG_LUAD were similar to those for TCGA‐LUAD, that is, 0.67, 0.75, and 0.63, respectively (Figure 6F). These results demonstrated that the predictive model built based on genes we identified in a cohort of predominantly Caucasian patients is prognostic in East Asian LUAD patients.

## 4 DISCUSSION

Age has been associated with survival in many cancer types.^38^, ^39^, ^40^, ^41^, ^42^, ^43^, ^44^ A recent systematic review of pan‐cancer prognostic clinicopathological factors associated with survival outcomes found that advanced patient age (135 studies) and higher pathological stage (133 studies) were common features associated with death from cancer, including LUAD.^45^ Previous studies have shown that aging and cancer are characterized by a series of partially overlapping “hallmarks” including genomic instability, epigenetic alterations, and chronic inflammation.^46^, ^47^ Although other features of aging, such as cellular senescence, might suppress cancer development and progression,^48^ it appears that the agonistic effects outweigh the antagonistic effects, resulting in an inverse correlation of age and cancer survival. Agreeing with these observations from clinical studies, we observed decreased survival in patients >70 years compared to those ≤70 years in TCGA‐LUAD.

Several factors may contribute to this negative association of age and OS in cancer including the presence of comorbidities, delayed diagnosis, and differences in treatment selection and response. Since older patients often have other health conditions which reduce organ function and physiological reserves, they are more fragile and their expected lifespan is shorter than younger patients. For example, lower OS was found in older breast cancer patients due to concurrent non‐oncological causes in a cohort of 1580 Portuguese women.^49^ Age above 70 years and Charlson Comorbidity Index higher than three are also associated with reduced survival after radical cystectomy in a cohort of 334 bladder cancer patients.^50^ In addition, older patients may receive less aggressive treatments compared to younger patients due to concerns about treatment toxicity and overall health, which may result in lower efficacy in eradicating cancer cells.^43^, ^51^, ^52^, ^53^, ^54^ Moreover, older patients are less responsive to certain treatments than younger patients due to age‐related changes in the cancer molecular landscape as well as the physiological reserve and organ function of the patients.^55^ For example, in advanced NSCLC, the OS benefit of ICI‐based immunotherapy in patients younger than 55 is six times higher than that of patients older than 75,^56^ which may be attributed, at least in part, to the immune suppressive TME in older patients discovered in this study.

Interestingly, the progression free survival was not significantly different between the older and younger cohorts (data not shown). One possible explanation for this lack of association of PFS with age relates to heterogeneity in the intensity of treatments across the population. In clinical practice, older patients often receive less intensive therapies because of higher comorbidities and frailty, and less intensive therapy could adversely affect disease‐specific survival and progression free survival in the older patients. However, our data suggest that host factors, such as the immune response mounted by the host to the cancer, could underlie the ultimate difference in OS. Another intriguing observation is that age was not associated with OS in late‐stage LUAD patients. Studies have shown that the immune complexity of LUAD increases during cancer progression, leading to remarkable intratumor immunological heterogeneity.^57^ In addition, a comprehensive multi‐omics analysis of the early and late stages of LUAD revealed important differences in molecular alterations and regulatory pathways during cancer progression,^58^ including significant differences in immunophenotype, which may lead to differential predictive power of age on OS in early versus late stage LUAD.

We chose to stratify patients using an age cut‐off of 70 to distinguish young and old patients with LUAD based on the median age of diagnosis in the United States between 2016 and 2020 being 71 years (https://seer.cancer.gov/statfacts/html/lungb.html). This median age appears to be consistent worldwide. A recent study of 241 NSCLC patients in Europe likewise reported a median age of 70.5 years.^59^ Moreover, Wu et al. conducted a study in 2025 Chinese LUAD patients and revealed age‐dependent genetic underpinnings in LUAD using cut‐offs of ≤50 years old (young), 51–69 years old (intermediate) and ≥70 years old (aged).^60^ In that study, the frequencies of alterations in eight clinical actionable genes were significantly different in the youngest compared to the oldest patients depending on the gene. In addition, the median tumor mutational burden was significantly higher in aged patients compared with the youngest group.^60^ Finally, age ≥70 years is also one of the most important predictors for not recommending certain treatments such as surgery and chemotherapy.^61^, ^62^ Together, these findings provide justification for the use of 70 years as an age cut off in our study.

Our study suggests that LUAD patients older than 70 have a more immunosuppressive TME compared to younger patients. Tumors from the older patients expressed significantly higher levels of the immune checkpoint protein PD‐L2 and the immunosuppressive T‐cell receptor TIM3, and both of these are associated with decreased T‐cell function. Our findings are consistent with the observation that the most striking feature of immunosenescence due to aging is the decline in T‐cell function, leading to impaired immunity and reduced response to pathogens.^63^ In addition, tumor infiltrating macrophages, which have been shown to be mostly immunosuppressive,^64^ were significantly higher in the older patients to younger patients. Age‐associated immune alterations have been largely overlooked, and older patients are substantially underrepresented in cancer immunotherapy clinical trials.^65^ Our study provides strong evidence that age‐related changes in immune TME are associated with LUAD patient survival and should be considered when treating older patients with immunotherapy approaches. With an aging population worldwide and increased use of immunotherapeutics for cancer, patient age needs to be considered in future clinical trial designs to better understand expected treatment outcomes across the lifespan.

We found that tumor‐associated neutrophils (TANs) in the TME were significantly lower in older compared to younger LUAD patients. TANs are recruited to the tumor from the spleen and bone marrow, and several reports have documented decreased chemotactic responses and aberrant migration of neutrophils in response to a variety of stimuli in older compared to young hosts.^66^ For example, aged mice, compared to young mice, showed decreased neutrophil recruitment to cutaneous wound infections.^67^ It is possible that reduced chemotaxis contributes to the lower neutrophil infiltration in the older LUAD patients observed in this study. Because TANs are extremely dynamic cells that can be either tumor‐promoting or suppressing depending on the TME,^68^ further investigations on the state of neutrophils and the chemotactic signals in LUAD are needed to understand these differences between age groups. Similarly, the quantitative differences in memory B cells between age groups merits additional study. Nonetheless, our data strongly suggest that immune cell infiltration is significantly different in older LUAD patients compared to younger patients as shown by the significant association of immune scores with age and the significant differences of cell fractions of different immune subtypes in TME of patients in different age groups.

We observed considerable overlap when using six different bioinformatics tools to identify significant changes in immune subtype infiltration between age groups. Specifically, higher infiltrating CD4+ T cells in older patients was a consistent finding by three different tools, providing a strong rational to further investigate the role of CD4+ T cells in age‐associated immune function in LUAD. Our findings are consistent with a previous report that CD4+ T cells were increased in the peripheral blood of older lung cancer patients compared to their younger counterparts.^69^ Similar age‐associated increases in CD4+ T cells have been observed in other diseases. For instance, older MS patients (age >60) harbor abnormally increased frequencies of circulating CD4+ T cells with activated and cytotoxic effector profiles compared to younger patients.^69^ It would be interesting to determine the composition of the CD4+ T cell population in older versus younger LUAD patients as it has been shown that there are functionally distinct helper, regulatory, and cytotoxic subpopulations of CD4+ T cells which may contribute to LUAD TME in distinct ways.

The algorithms did produce some inconsistencies in determining the proportion of immune cells among different age groups. For example, decreased neutrophils and increased M1 macrophages were only detected using CIBERSORT. Similar inconsistencies between analysis platforms have been observed in other cancer types. For example, an mRNA expression‐based stemness index (mRNAsi) in hepatocellular carcinoma (HCC) is negatively associated with CD8+ T cells when the data are analyzed using XCELL, while a positive association is detected using CIBERSORT and MCPCOUNTER.^70^ In addition, a pyroptosis‐associated long non‐coding RNA signature is positively associated with CD8+ T‐cell infiltration in bladder cancer when analyzed using TIMER, whereas XCELL, MCPCOUNTER, and CIBERSORT analyses show a negative correlation.^71^ Our study highlights that analysis of immune subtypes using only one of the publicly available tools should be viewed with caution, and our findings suggest that multiple tools should be used to obtain more robust results and a comprehensive understanding of immune infiltration in TME regardless of cancer type.

The enrichment of immune transcripts in DEGs between LUAD tissues with high versus low immune scores confirms the validity of the ESTIMATE algorithm. These genes are involved in a diverse set of pathways implicated in the regulation of immune function. For example, genes functioning in B‐ and T‐cell receptor pathways are enriched in the DEGs together with those mediating PD‐L1/PD‐1 immune checkpoint function. Moreover, genes important in biological processes that play crucial roles in immune function by regulating various aspects of the immune response, including inflammation, immune cell migration, activation, and immune signaling, such as the chemokine pathway and cytokine–cytokine receptor interactions, are also enriched in DEGs between LUAD tissues with high versus low immune scores. Finally, genes associated with primary immunodeficiency and human immunodeficiency virus 1 (HIV‐1) infection are overrepresented in the DEGs suggesting that genes important in the immune system's development and functioning early in life may play a role in immune regulation of TME in LUAD patients.

Several genes in the 22‐gene prognostic gene list have been shown previously to predict outcomes in NSCLC. For example, FGA, an adverse risk marker in our model, has been shown to predict poor survival in lung squamous cell carcinoma (LUSC), the second common subtype of NSCLC, in multiple patient cohorts.^72^ VGF, which is downregulated in older patients as well as in patients with high immune infiltrates, has also been reported to predict poor outcome and has been shown to promote chemoresistance in lung cancers with neuroendocrine features^73^ VGF may therefore negatively affect patient survival through mediating age and immune infiltration associated changes in LUAD. Another poor prognostic factor in our model, IL17C, has been shown to promote tumor growth in NSCLC xenograft models after its upregulation by proline dehydrogenase.^74^ Moreover, several studies have identified SCG2 as an immune‐associated biomarker that predicts poor survival in LUAD.^75^, ^76^, ^77^ These genes could serve as candidate therapeutic targets for LUAD treatment.

Ideally, useful biomarkers should display AUCs >0.8 to be highly clinically useful. However, there are numerous examples of AUCs of 0.6–0.7 that provide clinically useful information. Serum prostate‐specific antigen (PSA) testing for prostate cancer, with an AUC <0.7, is widely used for screening and disease management.^78^ In addition, Li et al. constructed a ferroptosis‐related gene model to predict survival in TCGA‐LUAD and reported AUCs of 0.698, 0.71, and 0.73 for the 1‐, 3‐, and 5‐year OS, respectively.^79^ In addition, Zhou et al. developed a six‐gene prognostic signature using GEO datasets and achieved a AUC of 0.667.^80^ Moreover, a predictive model based on weighted expression levels of eight genes achieved AUCs of 0.706, 0.748, and 0.698 at 1‐, 3‐ and 5‐year follow‐up, respectively in a training LUAD dataset, and the performance decreased in an independent test set with AUCs of 0.647, 0.612, and 0.58 for 1‐, 3‐ and 5‐year survival.^81^ Given the complex association between age and immune function with survival, improved algorithms based on a deepened understanding of the biology of these interactions could improve model performance.

Taken together, we find that age is associated with OS of LUAD patients, and appears to be mediated, at least in part, through differences in immune infiltration in the TME in young and old patients. Interestingly, the degree of immune infiltration was not influenced by smoking, the most important risk factor of lung cancer associated with OS,^82^ suggesting that aging is more important in shaping the immune TME than smoking in LUAD. Further investigations are needed to better understand the underlying mechanisms of aging and immune system function in modulating LUAD survival.

## AUTHOR CONTRIBUTIONS

**Andrew Zhou:** Conceptualization (supporting); data curation (equal); formal analysis (equal); writing – original draft (equal). **Dalin Zhang:** Conceptualization (supporting); data curation (equal); formal analysis (equal); writing – original draft (equal). **Xiaoman Kang:** Data curation (supporting); formal analysis (supporting). **James D. Brooks:** Conceptualization (lead); funding acquisition (lead); writing – review and editing (lead).

## CONFLICT OF INTEREST STATEMENT

The authors have declared that no competing interests exist.

## ETHICS STATEMENT

Ethical approval was not required for this work.

## Supporting information


Supplemental Figure 1

Supplemental Table 1:

## Data Availability

The data that support the findings of this study are publicly available in The Cancer Genome Atlas (TCGA), the Proteomic Data Commons (PDC), and the European Genome‐phenome Archive (EGA) databases.
