# Supplementary figures and images for: Identification of age‐ and immune‐related gene signatures for clinical outcome prediction in lung adenocarcinoma
Source: Cancer Med. 2023 Jul 11;12(16):17475–90. doi: 10.1002/cam4.6330 (PMC10501266; doi:10.1002/cam4.6330)

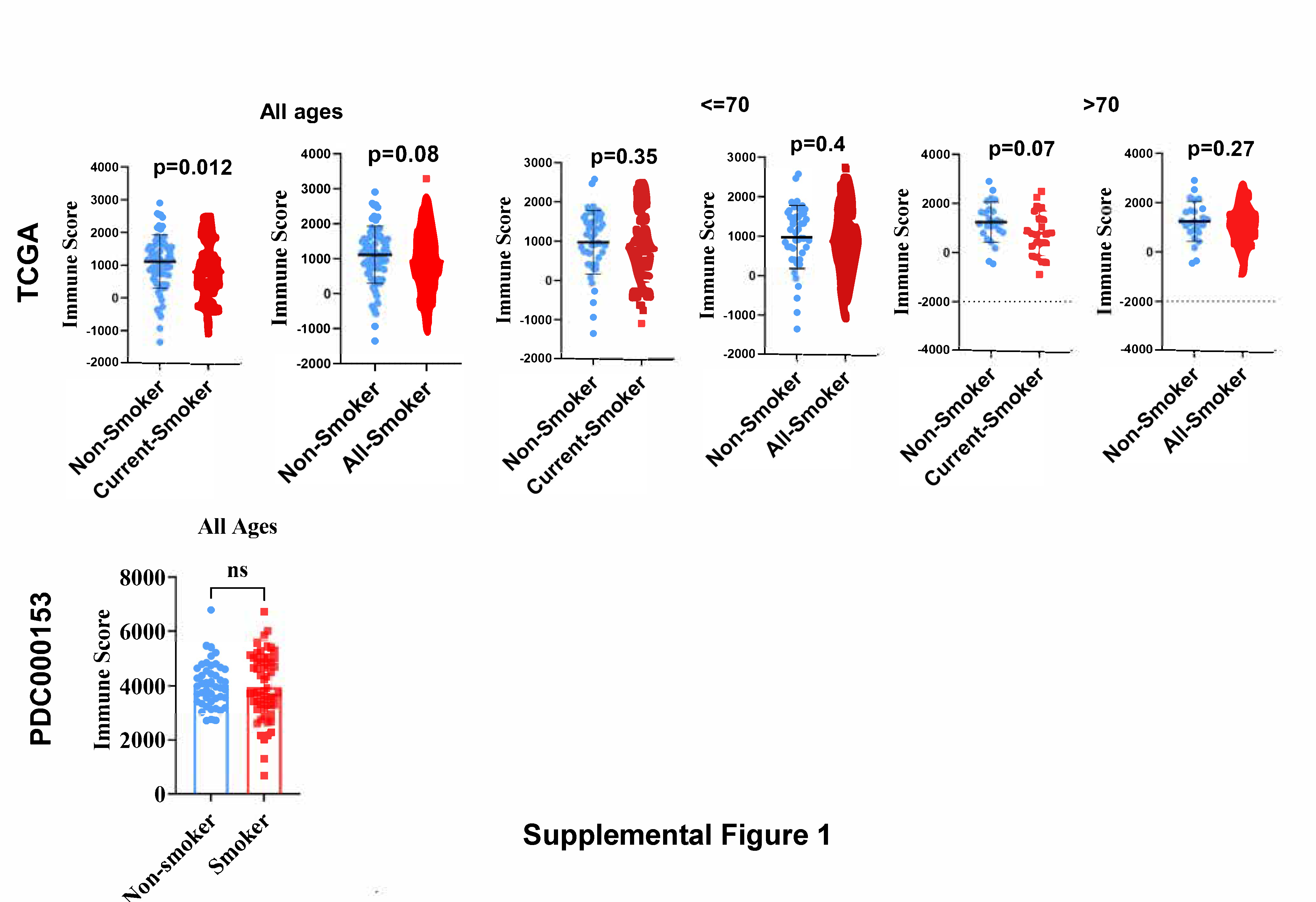

Supplement: Supplementary file 1 — Supplemental Figure 1 [file CAM4-12-17475-s001.tiff]
